# Sovereignty, equity, solidarity: progress on the Global Health Emergency Corps

**DOI:** 10.1136/bmjgh-2025-019424

**Published:** 2025-08-21

**Authors:** Scott F Dowell, Christophe Schmachtel, Abdi Mahamud, Abdou Salam Gueye, Andrew Lee, Armand Bejtullahu, Carita Davis, Ciro Ugarte, Didier K Ekouevi, Eduardo S Gudo, Flavio Salio, Gail Carson, Gina Samaan, Johanna Hanefeld, Linda Doull, Melkamu Abte, Michel Yao, Mohannad Al Nsour, Nedret Emiroglu, Nikki Romanik, Renee Christensen, Sadaf Lynes, Samar Al-Mutawakel, Shi Guoqing, Soha Shawqi Ahmed Albayat, Sugi Perera, Thebeyame Macheke, Valerie Nkamgang Bemo, Chikwe Ihekweazu, Michael J Ryan

**Affiliations:** 1 Health Emergencies, World Health Organization, Geneva, Switzerland; 2 Emergency Preparedness and Response Programme, Brazzaville, Congo; 3 UK Health Security Agency, London, UK; 4 Centre for Disease Control, Canberra, Canberra, Australia; 5 Pan American Health Organization, Washington, District of Columbia, USA; 6 Universite de Lome Faculte des Sciences de la Sante, Lome, Togo; 7 INSERM U1219 Bordeaux Population Health Research Université de Bordeaux, Bordeaux, France; 8 Instituto Nacional de Saúde, Maputo, Mozambique; 9 World Health Organisation, Headquarters, Geneva, Switzerland; 10 Tropical Medicine, Oxford University, Oxford, UK; 11 WHE, World Health Organization, Geneve, Switzerland; 12 Robert Koch Institut, Berlin, Germany; 13 Center of Public Health Emergency Management, Ethiopian Public Health Institute, Addis Ababa, Ethiopia; 14 World Health Organization Regional Office for Africa, Brazzaville, Congo; 15 Eastern Mediterranean Public Health Network, Amman, Jordan; 16 Brown University, Providence, Rhode Island, USA; 17 International Association of National Public Health Institutes (IANPHI), Brussels, Belgium; 18 WHO Eastern Mediterranean Regional Office, Cairo, Egypt; 19 Chinese Center for Disease Control and Prevention, Beijing, China; 20 Ministry of Public Health Qatar, Doha, Qatar; 21 Emergencies Department, WHO, Geneva, Switzerland; 22 Botswana Public Health Institute, Gaborone, Botswana; 23 Bill & Melinda Gates Foundation, Seattle, Washington, USA

**Keywords:** Global Health, Health policy, Public Health, COVID-19, Health Personnel

**SUMMARY BOX**

- The Global Health Emergency Corps (GHEC) offers a novel, structured approach to international crisis response by integrating national, regional and global experts through coordinated networks.
- It is defined as the body of experts in ministries and agencies in every country who work on health emergencies and the global ecosystem through which they coordinate.
- By prioritising sovereignty, equity and solidarity, GHEC seeks to overcome historical barriers to global health collaboration while respecting national autonomy in crisis response.
- GHEC leverages workforce pyramids and artificial intelligence, aiming to strengthen the emergency workforce, including surge capacities and leadership coordination.
- Sustained investment in GHEC can address gaps in global health governance, mitigate health emergencies more effectively and build trust across international health systems.

## Introduction

 Five years after the start of the COVID-19 pandemic, pushback against global health initiatives is common amidst declining health spending and growing nationalism.^1 2^ But the threat of future pandemics, such as that posed by the widening mammalian transmission of influenza A (H5N1), underscores the need for strengthened collaboration. The new Global Health Emergency Corps (GHEC) is a body of experts in ministries and agencies in every country who work on health emergencies and the global ecosystem through which they coordinate. The GHEC framework^3^ aims to support the development of consistent workforce capacity across the core health emergency components,^4^ complemented by surge teams that can be deployed as needed through pre-agreed governmental mechanisms, and emergency leaders who are embedded within the highest level of health security coordination in the government.

### GHEC’s promise: a new era of collaboration

The COVID-19 pandemic exposed significant weaknesses in global health response systems. As the Independent Panel on Pandemic Preparedness and Response stated, “Current institutions, public and private, failed to protect people from a devastating pandemic”.^5^ The panel called for a fundamental transformation to a coordinated response system, echoing WHO Director General Tedros Adhanom Ghebreyesus who had prioritised a strengthened global emergency workforce since the beginning of his first term,^6^ Bill Gates, who wrote about the need for a Global Epidemic Response and Mobilization team,^7^ and others.^8^ The GHEC promises to help make that transformation a reality.

Launched in 2023, GHEC integrates with the existing International Health Regulations as well as WHO’s updated Health Emergency Prevention, Preparedness, Response and Resilience framework,^4^ aiming to connect health emergency workforces, rapid response capacities and leaders in every country into a cohesive, proactive system. In addition, the newly endorsed Pandemic Agreement (articles 7, 16, 19) reinforces the importance of the GHEC by emphasising that each country should “develop, strengthen, and protect a skilled and adequate workforce to prevent, prepare for, and respond to health emergencies, including during pandemics …” GHEC, with its mandate for rapid response and global health expertise, serves as an operational arm of the Accord. It translates the high-level commitments of the Accord into action, providing the necessary surge capacity, technical expertise and coordination to support countries during health emergencies.

The approach to integration of GHEC with WHO regional and country offices, as well as existing networks and regional entities, was most recently highlighted by Exercise Polaris, a simulation held in April 2025 that brought these entities together with 15 participating countries to effectively control a pandemic from a fictional orthopox virus.^9^

### Vignette: how the GHEC could have changed the COVID-19 response

Had the GHEC existed in January 2020, the course of the COVID-19 pandemic might have played out differently. Corps members in countries around the world, having exercised scenarios such as the SARS-1 outbreak, could have enabled countries to put measures in place to contain the threat, as did many places with SARS-1 experience, such as Singapore, Vietnam, Canada, Thailand, Australia and China.^10^ SARS-CoV-2 was a more challenging pathogen, with pre-symptomatic transmission and biologically significant mutations, and the inequitable distribution of resources impeded control. Still, consistently applied measures, such as mask use, reduced gatherings and rigorous testing, along with effective community engagement, could have limited spread.^8 10^ The result could have been like the early COVID-19 results in those countries—delayed exponential growth of the pandemic. This flattening of the epidemic curve would have led to more results like the first SARS, with substantial containment of the pandemic threat. For places where the epidemic did manage to take hold, Corps members and their institutions, coordinated globally by WHO, might have concentrated efforts, sharing critical information and countermeasures. The pandemic could have been significantly curtailed earlier, potentially saving both lives and dollars.

### Strategic foundations: sovereignty, equity and solidarity

The GHEC framework is guided by three essential principles. Sovereignty recognises each nation’s autonomy to prioritise its citizens during a health emergency, manage the local response while fostering international collaboration. Equity explicitly addresses a shortcoming of the COVID-19 response in the availability of countermeasures where they are most needed to stop transmission and save lives.^8^ Solidarity recognises that an effective pandemic response also requires countries supporting each other by structuring similar emergency workforce pyramids, allowing for a more effective international response when needed.

Effective response to health emergencies also requires an ecosystem that builds on nationally mandated and interconnected emergency response institutions,^8^ aligns the workforce teams and leverages existing emergency response networks like the Global Outbreak Alert and Response Network (GOARN), the Emergency Medical Teams (EMT) network, the Global Health Cluster, the global network of Public Health Emergency Operations Centres (EOC-NET) and others.^4 11^ The GHEC framework recognises the invaluable collaborations established through these networks and adds value by advocating for consistency, facilitating resource sharing and optimising coordination. For example, a new deployment dashboard managed by GOARN tracks all deployments for Mpox including bilateral ones not managed by GOARN or WHO, and a newly established Global Health Emergency Corps unit at WHO demonstrates the commitment of the organisation to support countries in implementing the GHEC framework, leveraging the expertise of global health emergency networks like GOARN, EMT and EOC-NET whose secretariats are housed within the unit.

### GHEC: experts at the national level

The Emergency Corps is envisioned as a robust network of health emergency experts, primarily composed of national-level professionals, with smaller contingents at regional and global levels. This structure defers to national sovereignty while fostering international solidarity.

**Figure 1:**
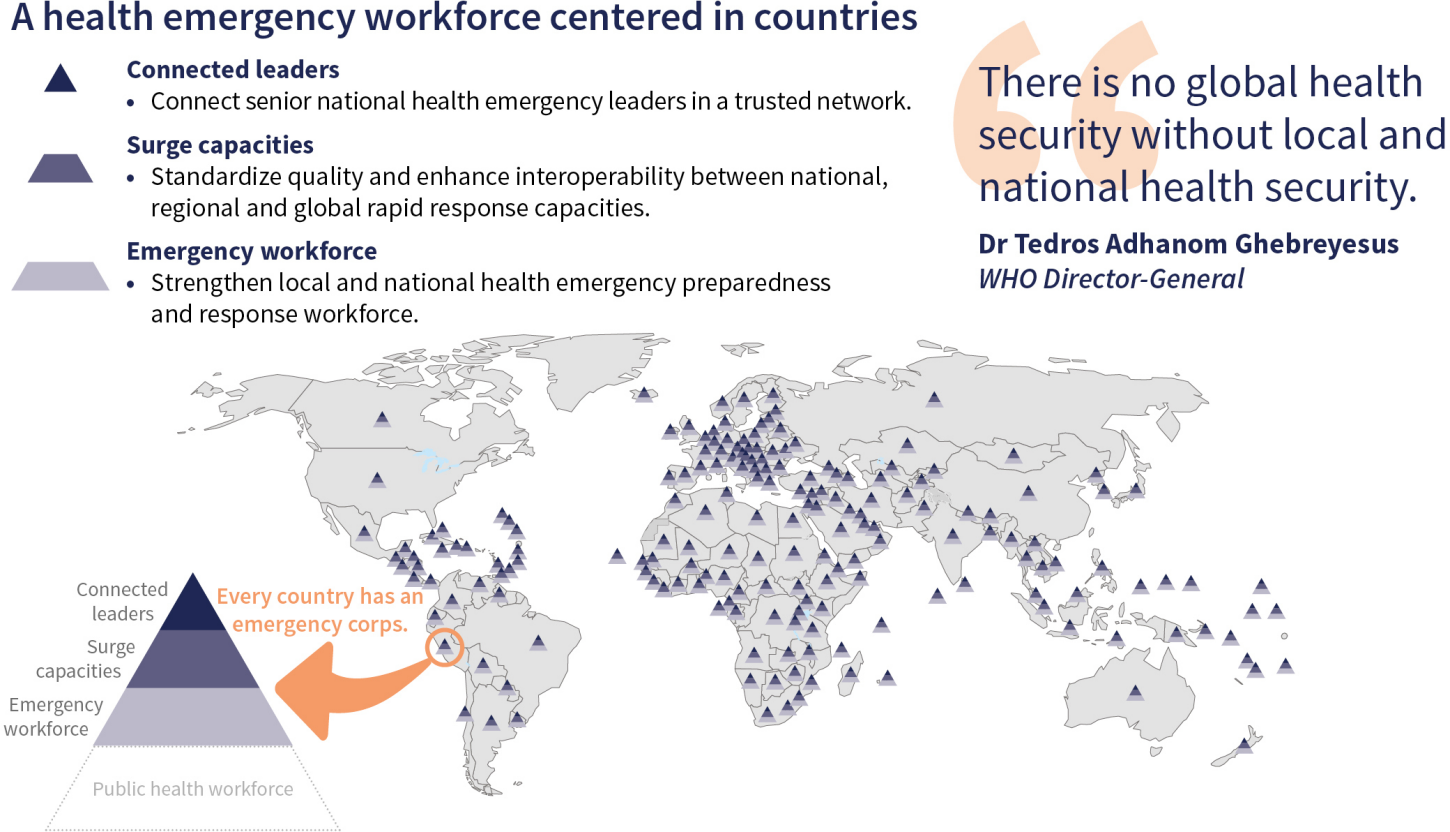
A Global Health Emergency Corps centred in every country.

In practice, each country is encouraged to adopt and adapt the GHEC framework, tailored to its specific institutions, laws, regulations and practices at national and sub-national levels. This will include identifying and investing in the health emergency workforce; well-trained surge teams and national health emergency leaders (figure 1). Together, these three levels can be visualised as a national Health Emergency Corps workforce pyramid that collaborates with the WHO country office and others as part of the country’s public health workforce. Budgetary support for this body of experts who work on domestic health emergencies has traditionally been the responsibility of countries themselves. With the establishment of the Pandemic Fund managed by the World Bank and other sources, there is increasing recognition that support for multinational emergencies and the ecosystem through which experts must coordinate should also be considered a global public good and supported as such, especially for low-income countries.

The health emergency workforce includes both dedicated staff and on-call expert responders. Dedicated staff maintain emergency preparedness and response capabilities, often operating from national Public Health Emergency Operations Centres.^12^ On-call emergency responders drawn from specialised areas such as clinical care, surveillance, community health workers and medical countermeasures can be mobilised quickly. The diverse sources of these experts, including governmental institutions, non-governmental organisations, universities and the private sector, necessitate clear institutional coordination mechanisms.

Deployable surge capacities are critical for managing health emergencies that may overwhelm local resources, especially in fragile health settings.^10 13^ Countries develop specialised surge staff and teams capable of rapid deployment.

Connected leaders at the national level help coordinate responses within their country and to multi-national emergencies.^8 14^ Each country ideally identifies technical leaders who advise policy makers, including heads of state in the event of a pandemic, about the most effective control measures in the critical early phases of an emergency. These leaders should be well-networked with trusted counterparts at national, regional and global levels through joint responses and simulation exercises.

The GHEC framework emphasises the importance of a well-coordinated national health emergency workforce that enables small outbreaks and other health emergencies to be controlled closest to where they start, deployable surge capacities to ensure no area is overwhelmed and connected leaders to facilitate a coordinated response to large emergencies and pandemics.

### GHEC: a well-connected regional and global ecosystem

**Figure 2:**
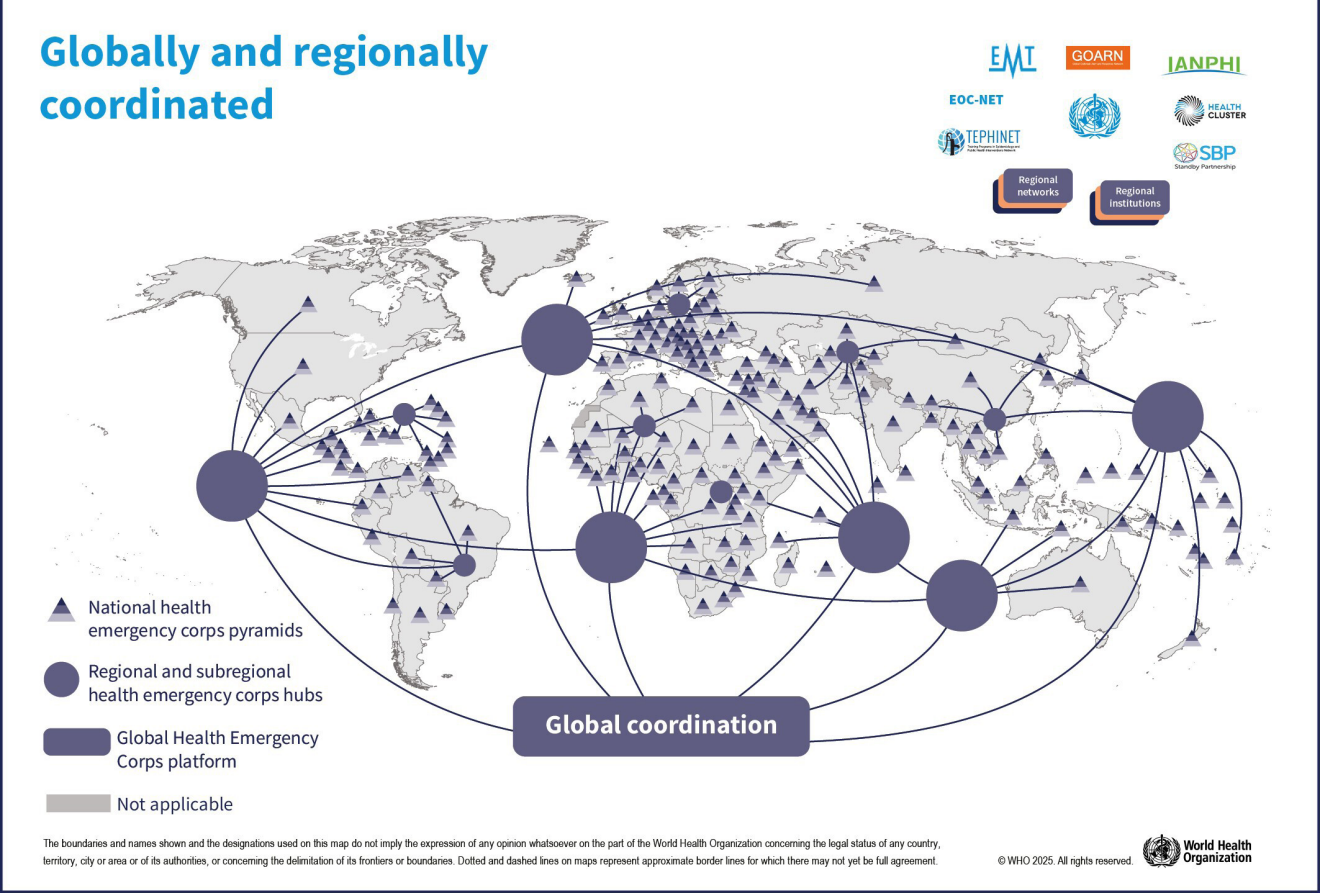
A regional and global ecosystem for coordination.

The GHEC is designed to enhance regional and global health emergency coordination (figure 2). The foundations for the GHEC ecosystem are Ministries of Health, national public health agencies or similarly mandated national institutions. This initiative also builds on the growing importance of regional organisations and the need for a cohesive global strategy.

Regional agencies have increasingly taken on critical roles in health emergency responses. The Africa CDC, European CDC and other entities have been pivotal in coordinating support to national responses, providing surge capacities and fostering networks of technical leaders.^13^ WHO Regional Emergency Operations Centers have been instrumental in managing crises like the H1N1 (2009) pandemic, Zika outbreak, cyclone Amphan and the West Africa Ebola outbreak.^15 16^ Regional entities have important roles in training and deploying surge teams, and financial support from programmes like EU4Health and grants from the World Bank bolster these efforts.^17 18^ Regional networks of technical leaders have been convened ad hoc in response to crises such as the Uganda Ebolavirus outbreak of 2022.^19^ GHEC encourages regional networks for joint advocacy and coordination. Leveraging advancements in artificial intelligence will also play a role in improving the basis of GHEC through optimised surveillance, workforce management and predictive analysis of health emergencies.

Global coordination is essential for managing multi-regional emergencies. A small group of experts within WHO headquarters’ incident management structure interfaces with regional and national structures. Global networks like GOARN, EMT and the Stand-by Partners mechanism coordinate the deployment of surge teams and experts. These established networks ensure interoperable responses to health emergencies and have been strengthened and interconnected in the post-pandemic period as promoted by GHEC. The newly established Global Health Emergency Corps unit at WHO headquarters committed to supporting countries in strengthening capacities of their own Health Emergency Corps and leveraging the collaboration through health emergency networks under the GHEC framework is an indication of the commitment to intraregional interoperability and organisational coherence.

### International collaboration against mpox clade 1b

In October 2024, WHO and its partners, in collaboration with Member States, activated the GHEC for the first time to support countries facing mpox clade 1b outbreaks.^20^ The activation followed the declaration of mpox as a public health emergency of international concern. 18 African countries had reported mpox cases, with the rapid spread of clade 1b raising concerns about further transmission.

Under the GHEC framework, WHO collaborated with the International Association of National Public Health Institutes to assess emergency workforce capacities in eight affected countries. The assessment identified 22 workforce gaps in areas such as epidemiology, surveillance and infection prevention. By mid-October, WHO had deployed 56 experts to the affected countries, including staff from the institutions in GOARN and the African Volunteers Health Corps (AVoHC-SURGE). Beyond deployment coordination, technical leaders from affected and previously affected countries were convened under the GHEC framework to consider the most effective control measures and share best practices. The impact of this initial activation of GHEC was modest, improving visibility of workforce gaps and coordinated deployments, but it highlighted the potential value for larger or more threatening epidemics.

## Conclusion

Building from a respect for national sovereignty while recognising the necessity for countries to coordinate in stopping a pandemic, GHEC is leveraging an increasing willingness of countries and partners around the world to collaborate in preparing for and responding to health emergencies. This body of experts from all countries who can work together in a connected ecosystem offers a more coordinated preparedness and response in health emergencies.

## Data Availability

There are no data in this work.
